# Carbohydrate Intake in Early Childhood and Body Composition and Metabolic Health: Results from the Generation R Study

**DOI:** 10.3390/nu12071940

**Published:** 2020-06-30

**Authors:** Anh N. Nguyen, Susana Santos, Kim V. E. Braun, Trudy Voortman

**Affiliations:** 1 Department of Epidemiology, Erasmus University Medical Center, 3000 CA Rotterdam, The Netherlands; a.n.nguyen@erasmusmc.nl (A.N.N.); k.braun@erasmusmc.nl (K.V.E.B.); 2 The Generation R Study Group, Erasmus University Medical Center, 3000 CA Rotterdam, The Netherlands; s.dasilvasantos@erasmusmc.nl; 3 Department of Pediatrics, Erasmus MC-Sophia Children’s Hospital, University Medical Center, 3000 CA Rotterdam, The Netherlands

**Keywords:** carbohydrates, macronutrients, childhood, infancy, obesity, cholesterol, cohort

## Abstract

High sugar intake in childhood has been linked to obesity. However, the role of macronutrient substitutions and associations with metabolic health remain unclear. We examined associations of carbohydrate intake and its subtypes with body composition and metabolic health among 3573 children participating in a population-based cohort in the Netherlands. Intake of total carbohydrate, monosaccharides and disaccharides, and polysaccharides at age 1 year was assessed with a food-frequency questionnaire. We repeatedly measured children’s height and weight to calculate BMI between their ages of 1 and 10 years. At ages 6 and 10 years, fat and fat-free mass were measured with dual-energy X-ray-absorptiometry and blood concentrations of triglycerides, cholesterol, and insulin were obtained. For all outcomes, we calculated age and sexspecific SD-scores. In multivariable-adjusted linear mixed models, we found no associations of intake of carbohydrates or its subtypes with children’s BMI or body composition. A higher intake of monosaccharides and disaccharides was associated with higher triglyceride concentrations (0.02 SDS per 10 g/day, 95% CI: 0.01, 0.04). Higher monosaccharide and disaccharide intake was also associated with lower HDL-cholesterol (−0.03 SDS, 95% CI: −0.04; −0.01), especially when it replaced polysaccharides. Overall, our findings suggest associations of higher monosaccharide and disaccharide intake in early childhood with higher triglyceride and lower HDL-cholesterol concentrations, but do not support associations with body composition.

## 1. Introduction

Dietary sugars have been suggested to be associated with several adverse health outcomes, including obesity and other metabolic risk factors. However, the effect of sugars on body weight has only been observed when a high sugar intake is accompanied by an excess energy intake: an isocaloric higher intake of sugars does not appear to affect weight, and current evidence for an effect of sugar intake on metabolic health is controversial [1,2,3]. Metabolic health factors, including dyslipidemia and insulin sensitivity, predict risk for type 2 diabetes and cardiovascular diseases in adults. Previous studies have suggested that several metabolic risk factors are already present in childhood and that these risk factors track into adulthood [4,5]. Therefore, it is important to study determinants of metabolic health already in childhood.

Although an isocaloric higher sugar intake does not appear to affect obesity, some studies suggest associations of intake of monosaccharides and disaccharides with insulin resistance and dyslipidemia in adults [6,7]. However, to interpret health effects of any dietary component, but especially for macronutrients, it is crucial to take into account effects of energy intake and overall macronutrient composition of the diet. A higher intake of one macronutrient in an isocaloric diet involves a lower intake of another macronutrient. Health effects may also differ for different substitutions. This has been shown for intake of for example fatty acids: a lower intake of saturated fat is associated with reduced triglycerides levels and lower cardiovascular risk when consumed at the expense of a higher polyunsaturated fat intake, but not when consumed at the expense of monounsaturated or trans-fat [8,9]. This suggests that associations of sugars with metabolic health may depend on the macronutrients that replace sugars in the diet. Indeed, a meta-analysis showed that the specific monosaccharide fructose only raised triglycerides levels in high doses or in isocaloric exchange for starch, but not for sucrose or a mix of carbohydrate sources [10].

Diet in early childhood may already affect cardiometabolic factors such as obesity or blood lipids. Since these cardiometabolic risk factors tend to track from childhood to adulthood, and increase the risk of cardiometabolic diseases, it is important to identify dietary factors involved. Previous studies mainly reported associations of sugars with weight or metabolic risk when restricted to sugar-sweetened beverages and not for sugar from other sources [11]. This suggests that, in addition to different macronutrient substitutions, different types of sugars (e.g., liquid versus solid) [12] may impact metabolic health differently. However, studies on the effect of total carbohydrate intake and its subtypes on metabolic risk factors in young children are scarce. Therefore, we aimed to examine associations of carbohydrate intake and its subtypes with metabolic health in a large population-based cohort of children. In addition, we examined the role of different macronutrient substitutions in these associations.

## 2. Methods

### 2.1. Study Design and Population

This study was embedded in the Generation R Study, an ongoing population-based cohort study from fetal life onward in Rotterdam, the Netherlands [13]. Pregnant women were enrolled between April 2002 and January 2006 and a total of 9749 live-born children were available for follow-up. Parents of all participating children provided written informed consent and approval was obtained from the medical ethical committee of Erasmus University Medical Center, Rotterdam [13].

At the child’s age of 1 year, a questionnaire to assess diet in early childhood was sent to parents of 5088 children. Dietary data was available for 3629 of these children. Of this group, 3573 children had data on anthropometrics and 3122 on body composition available at one or more time points up to age 10 years. In addition, 2556 children had data available on blood lipids or insulin. The number of children included in the analyses therefore differs per outcome, ranging from 2548 for triglycerides and insulin to 3573 for BMI.

### 2.2. Dietary Assessment

Dietary intake in early childhood was assessed using a validated semi-quantitative food-frequency questionnaire (FFQ) at the child’s age of 12.9 (12.7–19.9) months, as described in detail previously [14,15]. The FFQ was filled out by the caregivers and consisted of 211 food items and included questions on the frequency of consumption, serving sizes, type of food items, and preparation methods, covering the last month. Information on frequencies and quantities were converted into grams per day based on standard portion sizes. Intakes of nutrients were calculated using the Dutch Food Composition Table. Validation of the FFQ against three 24-h recalls in a representative sample of 32 Dutch children showed moderate to good ranking for intakes of nutrients [14,15].

### 2.3. Metabolic Health Measurements

Children’s anthropometrics were measured at eight different time points between ages 1 year and 10 years. All measurements were performed without shoes and heavy clothing. Up to age 4 years, measurements were taken during routine visits at the Child Health Centers at median ages of 14.3 (IQR 14.1–14.6), 18.3 (IQR 18.1–18.9), 24.7 (IQR 24.2–25.6), 30.5 (IQR 30.1–31.3), 36.6 (IQR 36.2–37.4), and 45.8 (IQR 45.3−46.6) months. At the ages of 6.0 (IQR 5.8–6.2) and 9.7 (IQR 9.6–9.8) years, measurements were performed during visits to our research center at Erasmus University Medical Center. Weight was measured with a mechanical personal scale (SECA, Almere, The Netherlands), and height was measured with a Harpenden stadiometer (Holtain Limited, Dyfed, UK). During these visits, we also measured body composition (fat and lean mass) with a DXA scanner (iDXA, Ge-Lunar, 2008, Madison, WI, USA) using enCORE software version 13.6. We calculated BMI (weight(kg)/height(m)^2^), fat mass index (FMI) (fat mass(kg)/height(m)^2^), and fat-free mass index (FFMI) (fat-free mass(kg)/height(m)^2^). In addition, non-fasting blood samples were obtained and concentrations of triglycerides, total cholesterol, HDL cholesterol, and insulin were determined with enzymatic methods (using a Cobas 8000 analyzer; Roche, Almere, The Netherlands). Concentrations of LDL cholesterol were calculated according to the Friedewald formula. We calculated age and sex specific standard deviation scores (SDS) for all outcomes based on available data from participants in the Generation R Study.

### 2.4. Covariates

Information on child’s date of birth, sex, and birth weight was obtained from medical records. Child’s ethnicity (Dutch; non-Dutch) was defined based on the country of birth of the parents, on which information was obtained with questionnaires. Information on breastfeeding was obtained for the first 4 months of life (never; partially; exclusively) via postnatal questionnaires. Information on child’s sport participation (yes; no) and screen time, defined as time spend watching television or computer use (<2; ≥2 h/day), was obtained using a questionnaire at the age of 6 years. Dietary intake of children was assessed at the age of 8 years and we calculated their diet quality score, reflecting adherence to age-specific dietary guidelines [16].

Information on maternal age, maternal educational level (low; high), folic acid supplement use in early pregnancy (no; started in first ten weeks; started periconceptional), and household income (<2200; ≥2200 Euros/month) was obtained using questionnaires at enrollment in the study. During each trimester, questionnaires were used to assess whether mothers drank alcohol (never; until pregnancy was known; continued drinking occasionally; continued drinking frequently) or smoked (never; until pregnancy was known; continued smoking during pregnancy). Maternal height and weight were measured at our research center at enrollment, and BMI was calculated.

### 2.5. Statistical Analyses

We were interested in the effect of carbohydrate intake independent of its energy content, therefore we used the nutrient residual method to adjust carbohydrate intake for total energy intake. Briefly, linear regressions with total energy intake as independent variable and carbohydrate intake as dependent variable were used to calculate energy-adjusted intakes of carbohydrate. To enhance interpretability, predicted carbohydrate intake for the mean energy intake was added as a constant.

Linear mixed models were used to examine associations of carbohydrate intake at age 1 year with trajectories of BMI between ages 1 and 10 years and body composition or metabolic factors between ages 6 and 10 years. This method incorporates all available repeated measurements of the outcomes simultaneously and takes into account that these measurements are correlated within participants. We used likelihood ratio tests to determine a suitable random effect structure, which we used in each of the longitudinal models. Covariates were selected based on previous literature or a 10% change in effect estimate. All associations were analyzed using 2 models: model 1 included sex, ethnicity, age at dietary assessment, and total energy intake; and model 2 was additionally adjusted for breastfeeding, birth weight, screen time, sports participation, household income, maternal educational level, maternal age, maternal BMI, smoking during pregnancy, and folic acid supplements. For associations of carbohydrate intake with blood lipids and insulin, an extra model was examined; model 3 was additionally adjusted for FMI and FFMI to examine whether these associations were independent of child’s body composition. To examine whether carbohydrate intake modified trajectories of growth and body composition, we included interactions between carbohydrate intake and age of outcome measurements in the fixed effects structure of the linear mixed models. Furthermore, to examine whether associations of carbohydrate intake with metabolic health and body composition differed by child’s sex, an interaction term was included in the models.

To explore different macronutrient substitution effects, we used multivariable nutrient density models. First, to examine associations of carbohydrate intake with metabolic health at the expense of either dietary fat or protein, we added either intake of dietary fat or protein in the abovementioned models. All nutrients entered in the model per 5 energy percent (E%). This method allows us to test whether associations of carbohydrate intake differed when carbohydrate intake was replaced by either dietary fat or protein intake. Second, to examine the association of monosaccharide and disaccharide intakes with metabolic health at the expense of polysaccharide and vice versa, we added intakes of dietary fat, protein, and either intake of polysaccharide or monosaccharide and disaccharide in the same model.

Because the FFQ was originally developed for Dutch children, we performed sensitivity analyses restricted to participants with a Dutch ethnic background. We also repeated our analyses after excluding post term infants (gestational age ≥ 42 weeks) (*n* = 255). In addition, we examined whether associations of diet in early childhood with metabolic health are independent of diet in mid childhood, by additionally adjusting for their diet quality score at age 8 years. To reduce potential bias due to missing values on covariates, these variables were multiple imputed (m = 10 imputations). We present pooled regression coefficients of the 10 imputed datasets. Results were considered statistically significant at *p* < 0.05, two-sided alpha error. The statistical analyses were carried out using R version 3.4.1 (The R Foundation for Statistical Computing, Vienna, Austria).

## 3. Results

### 3.1. Population Characteristics

Characteristics of the children and their mothers are presented in Table 1. Around half of the children were girls (51.0%) and the majority of the children had a Dutch ethnic background (68.2%). Total carbohydrate intake at the age of 12.9 months was 191.7 ± 58.9 g/day, which corresponds with 58.3 ± 6.1 E%. This is in line with recommendations for this age group [17]. Mean intakes of monosaccharide and disaccharide was 113.0 ± 40.9 g/day and mean polysaccharide intake was 77.5 ± 27.3 g/day. Major food sources of carbohydrates were bread and other grain products; sweet snacks (i.e., candy, cookies, candy bars); and sugar-containing beverages.

### 3.2. Associations of Carbohydrate Intake with Body Composition

Results from linear mixed models for diet at age 1 year with body composition up to age 10 years are presented in Table 2. Total carbohydrate intake or intake of monosaccharides and disaccharides or of polysaccharide at age 1 year were not associated with any of the longitudinal body composition outcomes up to age 10 years when consumed at the expense of either protein or fat (i.e., any other energy source, a specific substitution was not specified in the model). In additional substitution models, higher total carbohydrate intake consumed at the expense of specifically protein, not fat (i.e., fat entered as a constant in the model), showed associations with lower BMI and FMI (Supplemental Table S1). This was the case for total carbohydrate intake as well as its subtypes, all specifically when substituting protein.

### 3.3. Associations of Carbohydrate Intake with Metabolic Health

Results for associations with blood lipids and insulin levels are presented in Table 3. After adjustment for confounders (model 2), we observed that a higher total carbohydrate intake at age 1 year was associated with higher levels of triglyceride (0.02 SDS per 10 g/day higher carbohydrate intake, 95% CI: 0.01, 0.04). This association did not attenuate and remained statistically significant after additional adjustment for child’s body composition (model 3: 0.02 SDS, 95% CI: 0.01, 0.04). No associations of total carbohydrate intake with other metabolic outcomes were observed.

When further exploring subtypes of carbohydrate intake, we observed that the associations of total carbohydrate intake with triglycerides was driven by an association of monosaccharide and disaccharide intake with triglyceride concentrations (model 3: 0.02 SDS, 95% CI: 0.01, 0.04). Higher intake of monosaccharides and disaccharides was also associated with lower HDL cholesterol (model 3: −0.03 SDS, 95% CI: −0.04, −0.01). Polysaccharide intake was not associated with any of the outcomes. We did not observe any association of carbohydrate intake with total or LDL cholesterol, or with insulin levels.

In substitution models (Supplemental Table S2), we observed that associations of higher monosaccharide and disaccharide intake with higher triglyceride levels were approximately similar when it substituted either fat (0.05 SDS per 5 energy %, 95% CI 0.02, 0.09), protein (0.07 SDS, 95% CI: −0.003, 0.15), or polysaccharides (0.04 SDS, 95% CI: 0.01, 0.08). In line with this, higher polysaccharide intake was associated with lower triglyceride levels when consumed at the expense of monosaccharides and disaccharides. Associations of higher monosaccharide and disaccharide intake with lower HDL cholesterol were observed especially when it replaced polysaccharides (−0.05 SDS, 95% CI: −0.09, −0.02).

### 3.4. Additional Analyses

Associations did not differ between boys and girls (interactions of carbohydrate intake with sex, all *p* > 0.05). Sensitivity analyses restricted to children with a Dutch ethnic background showed similar effect estimates as observed in the whole population (Supplemental Tables S3 and S4), although in this subgroup, associations of monosaccharide and disaccharide intake with HDL cholesterol were no longer statistically significant (Supplemental Table S4). Sensitivity analyses in which we excluded post term infants also showed similar associations as observed in the whole study population (data not shown). Additional adjustment for diet quality at the age of 8 years did not affect our findings (data not shown).

## 4. Discussion

Results from this large population-based study among young children suggest that higher intake of carbohydrate or its subtypes in early childhood is not associated with body composition. Only when a higher carbohydrate intake specifically substituted protein in the diet, a lower BMI and body fat were observed, which is in line with what is known about adipogenic effects of high protein in infancy. We observed associations of higher intake of monosaccharides and disaccharides in early childhood with higher triglyceride levels and lower HDL cholesterol levels up to age 10 years, independent of body composition. No associations were observed for intake of total carbohydrate, polysaccharides, or monosaccharides and disaccharides with total or LDL cholesterol, or with insulin levels.

Previous studies mainly reported associations of sugar intake with body weight or metabolic risk when restricted to intake of sugar-sweetened beverages and not for sugar from other sources. A systematic review and meta-analysis by Malik et al. (2012) showed that intake of sugar-sweetened beverages is associated with weight gain both in children and adults [18]. This has also been reported by another systematic review [19]. However, in this latter systematic review, no associations of intake of other sugars with adiposity in children were found [19]. In line with this, we did not observe any associations of total carbohydrate intake or its subgroups with body composition in children, but we previously did observe in our study population that a higher intake of sugar-containing beverages in early childhood was associated with a higher BMI [20]. In our study population, main sources of total carbohydrate intake, but also of monosaccharide and disaccharide, were bread and breakfast products rather than sugar-containing beverages, which may explain our null-associations for intake of total carbohydrates or sugars with body composition. Combined, these results support previous findings that associations of sugar intake with weight or adiposity may be mainly driven by sugar-containing beverages and not by other types of sugar or carbohydrates when consumed in an isocaloric diet.

Additionally, for metabolic outcomes, most previously reported associations have been attributed to sugar-containing beverages rather than sugar or carbohydrates in general. For example, in previous analyses in the Generation R Study, we observed associations of a higher intake of sugar-containing beverages with a higher cardiometabolic risk factor score in young children, although not with any of the individual metabolic risk factors (body fat percentage, blood pressure, HDL-cholesterol, triglycerides, and insulin) [21]. Another study in a multi-ethnic cohort of older children aged 8–15 years found that higher intake of sugar-sweetened beverages was associated with higher triglyceride concentrations and lower HDL cholesterol [22]. In our current study, no associations were observed for carbohydrate or its subtypes with total or LDL cholesterol or with insulin levels. However, we did observe associations of higher monosaccharide and disaccharide intake in early childhood with higher triglyceride levels and lower HDL cholesterol levels. In macronutrient substitution models, these associations appeared not to be driven by substitution for one specific macronutrient in particular, but statistical significance was only reached in models where monosaccharides and disaccharides replaced fat or polysaccharides. Given that metabolic risk factors may track into adulthood, further studies with repeated dietary assessment in early childhood and with longer follow-up should examine whether these differences in blood lipid levels track into later childhood an adulthood.

For body composition, although intake of total carbohydrate or its subtypes was not associated with body composition when consumed at the expense of any other energy source (i.e., protein and/or fat unspecified), substitution models in which higher total carbohydrate intake replaced specifically protein intake, but not fat, showed associations with lower BMI and FMI in children. This finding is in line with previous studies on protein intake in early childhood. Results from both observational studies [23,24,25] and randomized controlled trials [26,27] showed that a lower protein intake in early childhood—combined with either a higher carbohydrate and/or a higher fat intake—is associated with lower body weight and BMI in children. These findings suggest that a high protein intake in early childhood should be discouraged.

Important strengths of this study are its prospective population-based cohort design and the large number of participants being studied. In addition, we had detailed measurements of body composition, which were measured with DXA, and we had information on many potential child and parental confounders. The extensive FFQ that we used to assess diet in early childhood was specifically developed for young children and from which we could calculate intake of macronutrients and its subtypes. A limitation of FFQs in general is that they are subject to measurement errors. By adjusting for total energy intake, we aimed to reduce this error. In addition, we applied substitution models, which allowed us to account for the effect of energy intake, but also for substitution effects of macronutrients in the diet. Using this approach, we were able to examine whether associations were due to a higher carbohydrate intake or a lower intake of another macronutrient. Although we were able to control for several socioeconomic and lifestyle factors, some of these factors may not have been measured perfectly and we could have missed some important factors. For example, we did not have detailed information on physical activity. Therefore, we used sports participation as a proxy, which could have led to residual confounding. In addition, the use of non-fasting blood samples may have resulted in non-differential misclassification of the metabolic factors and may therefore have led to an underestimation of the observed associations. Finally, most of the participants included in our study had a Dutch ethnic background, had parents who were highly educated, and had a high household income, which may limit the generalizability of our findings to other populations.

## 5. Conclusions

In conclusion, our findings do not support that intake of carbohydrates or its subtypes in early childhood is associated with body composition, but do suggest that a higher carbohydrate intake in infancy, especially monosaccharides and disaccharides, may be associated with higher triglyceride levels and lower HDL cholesterol levels—independent of body composition. Further studies with longer follow-up are needed to explore whether this has consequences for later childhood and adulthood, taking into account macronutrient substitution effects.

## Figures and Tables

**Table 1:** Characteristics of the children and their mothers (*n* = 3573).

	Mean, Median, or %
**Child characteristics**	
Sex, girls	51.0
Ethnicity, Dutch	68.2
Birth weight (g)	3446 ± 580
Breastfeeding	
Exclusively ≥4 months	28.0
Partially ≥4 months	63.0
Never	9.0
**Child characteristics at dietary assessment**	
Age at FFQ (months)	12.9 (12.7–19.9)
Total energy intake (kcal)	1321 ± 413
Carbohydrate intake (g/day)	191.7 ± 58.9
Carbohydrate intake (E%/day)	58.3 ± 6.1
Monosaccharide and disaccharide intake (g/day)	113.0 ± 40.9
Monosaccharide and disaccharide intake (E%/day)	34.2 ± 6.9
Polysaccharide intake (g/day)	77.5 ± 27.3
Polysaccharide intake (E%/day)	23.8 ± 5.5
**Child characteristics at 6-year-visit**	
Age (years)	5.9 (5.8–6.0)
Sports participation, yes	43.8
Screen time, ≥2 h/day	24.2
Height (cm) (*n* = 2984)	118.2 ± 5.2
Weight (kg) (*n* = 2984)	21.8 (20.2−24.0)
Body mass index (kg/m^2^) (*n* = 2984)	15.7 (15.10–16.7)
Fat mass index (kg/m^2^) (*n* = 2911)	3.6 (3.1–4.3)
Fat-free mass index (kg/m^2^) (*n* = 2911)	11.9 (11.3–12.5)
Triglyceride (mmol/L) (*n* = 2003)	0.97 (0.72–1.29)
Total cholesterol (mmol/L) (*n* = 2007)	4.22 ± 0.63
LDL cholesterol (mmol/L) (*n* = 2008)	2.37 ± 0.56
HDL cholesterol (mmol/L) (*n* = 2008)	1.34 ± 0.46
Insulin (pmol/L) (*n* = 1998)	115 (64–187)
**Maternal characteristics**	
Maternal age (years)	31.4 ± 4.6
Maternal educational level, high	63.0
Maternal body mass index (kg/m^2^)	23.5 (21.6–26.2)
Smoking during pregnancy	
Never	77.6
Until pregnancy was known	10.0
Continued	12.4
Alcohol consumption during pregnancy	
Never	38.4
Until pregnancy was known	14.5
Continued occasionally	38.0
Continued occasionally frequently	9.2
Folic acid supplementation during pregnancy	
Started periconceptional	52.5
Started first 10 weeks	31.2
None	16.2
Household income, ≥2200 Euros/month	66.4

Values are means ± SD for continuous variables with a normal distribution, medians (interquartile range) for continuous variables with a skewed distribution, or percentages for categorical variables; based on imputed data (*n* = 10 imputations).

**Table 2:** Associations of carbohydrate intake at age 1 year with body composition up to age 10 years.

	BMI (SDS) (*n =* 3573)	FMI (SDS) (*n =* 3112)	FFMI (SDS) (*n =* 3112)
**Total carbohydrate intake (10 g/day)**			
Model 1	−0.01 (−0.02, 0.01)	−0.01 (−0.02, 0.004)	0.003 (−0.01, 0.02)
Model 2	−0.004 (−0.02, 0.01)	−0.01 (−0.02, 0.01)	0.005 (−0.01, 0.02)
**Total monosaccharide and disaccharide intake (10 g/day)**			
Model 1	0.004 (−0.01, 0.02)	0.002 (−0.01, 0.01)	0.003 (−0.01, 0.02)
Model 2	0.002 (−0.01, 0.01)	0.002 (−0.01, 0.01)	0.01 (−0.01, 0.02)
**Total polysaccharide intake (10 g/day)**			
Model 1	−0.01 (−0.03, 0.004)	−0.01 (−0.03, 0.004)	−0.004 (−0.02, 0.01)
Model 2	−0.01 (−0.02, 0.01)	−0.01 (−0.02, 0.01)	0.000 (−0.02, 0.02)

Values are regression coefficients and 95% confidence intervals based on linear mixed models reflect differences in body composition (age and sex specific SD-scores) per 10 g/day higher energy-adjusted carbohydrate intake when consumed at the expense of protein and/or fat. Model 1 (basic) is adjusted for sex, ethnicity, age at dietary assessment, and total energy intake. Model 2 (confounder) is additionally adjusted for breastfeeding, birth weight, screen time, sports participation, household income, maternal educational level, maternal age, maternal BMI, smoking during pregnancy, and folic acid supplements. Corresponding macronutrient substitution models are presented in Supplemental Table S1.

**Table 3:** Associations of carbohydrate intake at age 1 year with metabolic health up to age 10 years.

	Triglycerides (SDS) (*n =* 2548)	Total Cholesterol (SDS) (*n =* 2554)	HDL-Cholesterol (SDS) (*n =* 2556)	LDL-Cholesterol (SDS) (*n =* 2554)	Insulin (SDS) (*n =* 2548)
**Total carbohydrate intake (10 g/day)**					
Model 1	**0.02 (0.004, 0.04)**	0.001 (−0.02, 0.02)	−0.01 (−0.03, 0.01)	0.001 (−0.02, 0.02)	−0.002 (−0.02, 0.01)
Model 2	**0.02 (0.01, 0.04)**	0.001 (−0.02, 0.02)	−0.02 (−0.03, 0.003)	0.002 (−0.02, 0.02)	−0.002 (−0.02, 0.01)
Model 3	**0.02 (0.01, 0.04)**	0.002 (−0.02, 0.02)	−0.02 (−0.04, 0.001)	0.004 (−0.01, 0.02)	−0.001 (−0.02, 0.01)
**Total monosaccharide and disaccharide intake (10 g/day)**					
Model 1	**0.02 (0.01, 0.04)**	−0.000 (−0.02, 0.02)	**−0.03 (−0.04, −0.01)**	0.01 (−0.01, 0.03)	−0.01 (−0.02, 0.01)
Model 2	**0.02 (0.01, 0.04)**	0.000 (−0.02, 0.02)	**−0.03 (−0.04, −0.01)**	0.01 (−0.01, 0.03)	−0.01 (−0.02, 0.01)
Model 3	**0.02 (0.01, 0.04)**	0.000 (−0.01, 0.02)	**−0.03 (−0.04, −0.01)**	0.01 (−0.01, 0.03)	−0.01 (−0.02, 0.01)
**Total polysaccharide intake (10 g/day)**					
Model 1	−0.01 (−0.03, 0.01)	0.002 (−0.02, 0.02)	0.02 (0.001, 0.04)	−0.01 (−0.03, 0.01)	0.01 (−0.01, 0.03)
Model 2	−0.01 (−0.03, 0.01)	0.001 (−0.02, 0.02)	0.02 (−0.000, 0.04)	−0.01 (−0.03, 0.01)	0.01 (−0.01, 0.03)
Model 3	−0.01 (−0.03, 0.01)	0.001 (−0.02, 0.02)	0.02 (−0.001, 0.04)	−0.01 (−0.03, 0.01)	0.01 (−0.004, 0.03)

Values are regression coefficients and 95% confidence intervals from linear regression models and reflect differences in metabolic outcomes (age-and sex specific SD-scores) per 10 g/day higher energy-adjusted carbohydrate intake. Bold values indicate statistically significant effect estimates. Model 1 (basic) is adjusted for sex, ethnicity, age at dietary assessment, and total energy intake. Model 2 (confounder) is additionally adjusted for breastfeeding, birth weight, screen time, sports participation, household income, maternal educational level, maternal age, maternal BMI, smoking during pregnancy, and folic acid supplements. Model 3 (body composition) is additionally adjusted for FMI and FFMI. Corresponding macronutrient substitution models are presented in Supplemental Table S2.

## Supplementary Materials

The following are available online at https://www.mdpi.com/2072-6643/12/7/1940/s1. Table S1: Associations of carbohydrate intake at the expense of other macronutrients at the age of 1 year with body composition up to age 10 years, Table S2: Adjusted associations of carbohydrate intake, at the expense of other macronutrients at the age of 1 year with metabolic health up to age 10 years, Table S3: Associations of carbohydrate intake at age 1 year with body composition up to age 10 years in children with a Dutch ethnic background only, Table S4: Associations of carbohydrate intake at age 1 year with metabolic health up to age 10 years in children with a Dutch ethnic background only.
