# Supplementary material for: Carbohydrate Intake in Early Childhood and Body Composition and Metabolic Health: Results from the Generation R Study
Source: Nutrients. 2020 Jun 30;12(7):1940. doi: 10.3390/nu12071940 (PMC7399886; doi:10.3390/nu12071940)
Supplement: Supplementary file 1 [file nutrients-12-01940-s001.pdf]

SUPPLEMENTAL MATERIAL

Carbohydrate Intake in Early Childhood and Body composition and Metabolic Health: Results from the Generation R Study

Nguyen, Santos, Braun, Voortman

Erasmus University Medical Center Rotterdam, the Netherlands

Correspondence to: trudy.voortman@erasmusmc.nl

Table S1. Associations of carbohydrate intake at the expense of other macronutrients at the age of 1 year with body composition up to age 10 years

	**BMI (SDS)** ***(n=3,573)***	**FMI (SDS)** ***(n=3,112)***	**FFMI (SDS)** ***(n=3,112)***
**Total carbohydrate intake (5E%)**			
5E% lower fat	0.01 (-0.01, 0.04)	-0.003 (-0.03, 0.02)	0.02 (-0.01, 0.05)
5E% lower protein	**-0.14 (-0.19, -0.08)**	**-0.08 (-0.14, -0.02)**	-0.04 (-0.10, 0.03)
**Total monosaccharide and disaccharide intake (5E%)**			
5E% lower fat	0.02 (-0.01, 0.05)	0.01 (-0.02, 0.04)	0.02 (-0.01, 0.05)
5E% lower protein	**-0.14 (-0.20, -0.09)**	**-0.08 (-0.14, -0.02)**	-0.04 (-0.10, 0.03)
5E% lower polysaccharides	0.02 (-0.003, 0.05)	0.03 (-0.002, 0.05)	0.001 (-0.03, 0.03)
**Total polysaccharide intake (5E%)**			
5E% lower fat	-0.002 (-0.03, 0.03)	-0.02 (-0.05, 0.01)	0.02 (-0.02, 0,05)
5E% lower protein	**-0.16 (-0.23, -0.10)**	**-0.11 (-0.18, -0.04)**	-0.04 (-0.12, 0,04)
5E% lower monosaccharides	-0.02 (-0,05, 0.003)	-0.03 (-0.05, 0.002)	-0.001 (-0.03, 0.03)

Values are regression coefficients based on covariate-adjusted linear regression models and reflect differences in body composition (age- and sex specific SD-scores) per 5 E% higher intake of carbohydrate or its subtypes at the expense of either dietary fat or protein.

Table S2. Adjusted associations of carbohydrate intake, at the expense of other macronutrients at the age of 1 year with metabolic health up to age 10 years

	**Triglycerides (SDS)** ***(n=2,548)***	**Total cholesterol (SDS)** ***(n=2,554)***	**HDL-cholesterol (SDS)** ***(n=2,556)***	**LDL-cholesterol (SDS)** ***(n=2,554)***	**Insulin (SDS)** ***(n=2,548)***
**Total carbohydrate intake (5E%)**					
5E% lower fat	**0.04 (0.003, 0.07)**	0.01 (-0.03, 0.04)	-0.01 (-0.05, 0.02)	0.000 (-0.04, 0.04)	-0.002 (-0.03, 0.03)
5E% lower protein	**0.08 (0.01, 0.15)**	0.01 (-0.07, 0.09)	-0.01 (-0.09, 0.07)	0.001 (-0.08, 0.08)	0.01 (-0.06, 0.08)
**Total monosaccharide and disaccharide intake (5E%)**					
5E% lower fat	**0.05 (0.02, 0.09)**	-0.001 (-0.04, 0.04)	-0.03 (-0.07, 0.004)	0.003 (-0.04, 0.04)	-0.01 (-0.04, 0.02)
5E% lower protein	0.07 (-0.003, 0.15)	0.02 (-0.07, 0.10)	0.003 (-0.08, 0.08)	0.002 (-0.08, 0.08)	0.02 (-0.05, 0.09)
5E% lower polysaccharides	**0.04 (0.01, 0.08)**	-0.02 (-0.05, 0.02)	**-0.05 (-0.09, -0.02)**	0.01 (-0.03, 0.05)	-0.02 (-0.05, 0.01)
**Total polysaccharide intake (5E%)**					
5E% lower fat	0.01 (-0.03, 0.05)	0.02 (-0.03, 0.06)	0.02 (-0.02, 0,06)	-0.004 (-0.05, 0.04)	0.01 (-0.03, 0.05)
5E% lower protein	0.03 (-0.06, 0.12)	0.03 (-0.06, 0.13)	0.06 (-0.04, 0,15)	-0.01 (-0.10, 0.09)	0.04 (-0.04, 0.12)
5E% lower monosaccharides	**-0.04 (-0.08, -0.01)**	0.02 (-0.02, 0.05)	**0.05 (0.02, 0,09)**	-0.01 (-0.05, 0.03)	0.02 (-0.01, 0.05)

Values are regression coefficients based on covariate-adjusted linear regression models and reflect differences in metabolic outcomes (age- and sex specific SD-scores) per 5 E% higher intake of carbohydrate or its subtypes at the expense of either dietary fat or protein.

Table S3. Associations of carbohydrate intake at age 1 year with body composition up to age 10 years in children with a Dutch ethnic background only

	**BMI (SDS)** ***(n=2,437)***	**FMI (SDS)** ***(n=2,437)***	**FFMI (SDS)** ***(n=2,437)***
**Total carbohydrate intake (10g/d)**			
Model 1	0.01 (-0.01, 0.02)	0.01 (-0.01, 0.02)	-0.002 (-0.02, 0.02)
Model 2	0.002 (-0.01, 0.02)	-0.001 (-0.02, 0.01)	0.002 (-0.02, 0.02)
**Total monosaccharide and disaccharide intake (10g/d)**			
Model 1	0.001 (-0.01, 0.02)	0.01 (-0.002, 0.03)	-0.01 (-0.03, 0.004)
Model 2	-0.003 (-0.02, 0.01)	0.01 (-0.01, 0.02)	-0.01 (-0.03, 0.005)
**Total polysaccharide intake (10g/d)**			
Model 1	0.01 (-0.01, 0.03)	-0.01 (-0.03, 0.01)	0.02 (-0.003, 0,04)
Model 2	0.01 (-0.01, 0.03)	-0.01 (-0.03, 0.01)	0.02 (-0.001, 0,05)

Values are regression coefficients and 95% confidence intervals based on linear mixed models reflect differences in body composition (age- and sex specific SD-scores) per 10 grams/day higher energy-adjusted carbohydrate intake.

Model 1 (basic) is adjusted for sex, ethnicity, age dietary assessment, and total energy intake.

Model 2 (confounder) is additionally adjusted for breastfeeding, birth weight, screen time, sports participation, household income, maternal educational level, maternal age, maternal BMI, smoking during pregnancy, and folic acid supplements.

Table S4. Associations of carbohydrate intake at age 1 year with metabolic health up to age 10 years in children with a Dutch ethnic background only

	**TG (SDS)** ***(n=1,755)***	**Total cholesterol (SDS)** ***(n=1,759)***	**HDL-cholesterol (SDS)** ***(n=1,761)***	**LDL-cholesterol (SDS)** ***(n=1,758)***	**Insulin (SDS)** ***(n=1,757)***
**Total carbohydrate intake (10g/d)**					
Model 1	**0.03 (0.01, 0.05)**	0.01 (-0.01, 0.04)	-0.004 (-0.03, 0.02)	0.01 (-0.01, 0.04)	0.02 (-0.01, 0.02)
Model 2	**0.03 (0.01, 0.05)**	0.01 (-0.01, 0.04)	-0.003 (-0.03, 0.02)	0.01 (-0.01, 0.03)	0.02 (-0.01, 0.03)
Model 3	**0.03 (0.01, 0.05)**	0.01 (-0.01, 0.04)	-0.003 (-0.03, 0.02)	0.01 (-0.01, 0.03)	0.02 (-0.01, 0.03)
**Total monosaccharide and disaccharide intake (10g/d)**					
Model 1	**0.03 (0.01, 0,05)**	0.01 (-0.01, 0.03)	-0.02 (-0.04, 0.004)	0.02 (-0.01, 0.04)	0.001 (-0.02, 0.02)
Model 2	**0.03 (0.01, 0,05)**	0.01 (-0.01, 0.03)	-0.02 (-0.04, 0.01)	0.02 (-0.005, 0.04)	0.001 (-0.02, 0.02)
Model 3	**0.03 (0.01, 0,05)**	0.01 (-0.01, 0.03)	-0.01 (-0.03, 0.01)	0.01 (-0.01, 0.03)	0.001 (-0.02, 0.02)
**Total polysaccharide intake (10g/d)**					
Model 1	-0.01 (-0.04, 0.02)	0.01 (-0.02, 0.04)	0.03 (-0.002, 0,06)	-0.01 (-0.04, 0.02)	0.01 (-0.01, 0.04)
Model 2	-0.01 (-0.04, 0.02)	0.01 (-0.02, 0.04)	0.03 (-0.002, 0,06)	-0.01 (-0.04, 0.02)	0.01 (-0.01, 0.04)
Model 3	-0.01 (-0.04, 0.02)	0.01 (-0.02, 0.04)	0.02 (-0.01, 0,05)	-0.005 (-0.03, 0.03)	0.02 (-0.01, 0.04)

Values are regression coefficients and 95% confidence intervals from linear regression models and reflect differences in metabolic outcomes (age- and sex specific SD-scores) per 10 grams/day higher energy-adjusted carbohydrate intake. **Bold** values indicate statistically significant effect estimates.

Model 1 (basic) is adjusted for sex, ethnicity, age dietary assessment, and total energy intake.

Model 2 (confounder) is additionally adjusted for breastfeeding, birth weight, screen time, sports participation, household income, maternal educational level, maternal age, maternal BMI, smoking during pregnancy, and folic acid supplements.

Model 3 (body composition) is additionally adjusted for FMI and FFMI.
